# Production of 2,3-butanediol from glucose and cassava hydrolysates by metabolically engineered industrial polyploid *Saccharomyces cerevisiae*

**DOI:** 10.1186/s13068-019-1545-1

**Published:** 2019-08-29

**Authors:** Ye-Gi Lee, Jin-Ho Seo

**Affiliations:** Department of Agricultural Biotechnology and Center for Food and Bioconvergence, Seoul National University, Seoul, 08826 Republic of Korea

**Keywords:** Industrial yeast, Polyploid *Saccharomyces cerevisiae*, 2,3-Butanediol, CRISPR-Cas9, Cassava hydrolysate

## Abstract

**Background:**

2,3-Butanediol (2,3-BDO) is a valuable chemical for industrial applications. Bacteria can produce 2,3-BDO with a high productivity, though most of their classification as pathogens makes them undesirable for the industrial-scale production. Though *Saccharomyces cerevisiae* (GRAS microorganism) was engineered to produce 2,3-BDO efficiently in the previous studies, their 2,3-BDO productivity, yield, and titer were still uncompetitive compared to those of bacteria production. Thus, we propose an industrial polyploid *S. cerevisiae* as a host for efficient production of 2,3-BDO with high growth rate, rapid sugar consumption rate, and resistance to harsh conditions. Genetic manipulation tools for polyploid yeast had been limited; therefore, we engineered an industrial polyploid *S. cerevisiae* strain based on the CRISPR-Cas9 genome-editing system to produce 2,3-BDO instead of ethanol.

**Results:**

Endogenous genes coding for pyruvate decarboxylase and alcohol dehydrogenase were partially disrupted to prevent declined growth rate and C_2_-compound limitation. A bacterial 2,3-BDO-producing pathway was also introduced in engineered polyploid *S. cerevisiae*. A fatal redox imbalance was controlled through the heterologous NADH oxidase from *Lactococcus lactis* during the 2,3-BDO production. The resulting strain (YG01_SDBN) still retained the beneficial traits as polyploid strains for the large-scale fermentation. The combination of partially disrupted *PDC* (pyruvate decarboxylase) and *ADH* (alcohol dehydrogenase) did not cause the severe growth defects typically found in all pdc- or adh-deficient yeast. The YG01_SDBN strain produced 178 g/L of 2,3-BDO from glucose with an impressive productivity (2.64 g/L h). When a cassava hydrolysate was used as a sole carbon source, this strain produced 132 g/L of 2,3-BDO with a productivity of 1.92 g/L h.

**Conclusions:**

The microbial production of 2,3-BDO has been limited to bacteria and haploid laboratorial *S. cerevisiae* strains. This study suggests that an industrial polyploid *S. cerevisiae* (YG01_SDBN) can produce high concentration of 2,3-BDO with various advantages. Integration of metabolic engineering of the industrial yeast at the gene level with optimization of fed-batch fermentation at the process scale resulted in a remarkable achievement of 2,3-BDO production at 178 g/L of 2,3-BDO concentration and 2.64 g/L h of productivity. Furthermore, this strain could make a bioconversion of a cassava hydrolysate to 2,3-BDO with economic and environmental benefits. The engineered industrial polyploid strain could be applicable to production of biofuels and biochemicals in large-scale fermentations particularly when using modified CRISPR-Cas9 tools.

## Background

The emerging platform materials of C2–C4 diol isomers have been studied for production from renewable resources. Especially, 2,3-butanediol (2,3-BDO) is a promising chemical with various industrial applications [1]. It is used in the production of a wide range of needed derivatives, such as softening agents, plasticizer, polyester, drugs, and cosmetics [2, 3]. In addition, 2,3-BDO can be used as a starting material for chemical conversion, especially for production of 2-butanone (commonly methyl ethyl ketone or MEK) and 1,3-butadiene. MEK considered as an effective liquid-fuel additive and 1,3-butadiene used to produce the synthetic rubber are the major building blocks in the chemical industry [4, 5]. It has been attempted that the promising platform materials can be produced in the engineered microorganism to apply large-scale fermentation. For instance, a biotechnology company of Genomatica (San Diego, USA) constructed a commercial pipeline for producing 1,4-BDO as much as 30,000 ton/year via the engineered *Escherichia coli* [6]. The 2,3-BDO market need is expected to reach 74 kilotons by 2018 [7] and the estimated annual global market value of 2,3-BDO derivatives is approximately 43 billion dollars [4]. Thus, global demand of industrial-scale production of 2,3-BDO from biomass is increasing [8].

Petroleum-based chemical processes currently dominate commercial 2,3-BDO production [9]. Due to future global regulation of carbon dioxide emissions, scientists suggest that microbial production of 2,3-BDO could be a powerful alternative if the current low productivity and high production cost could be overcome. Microbial production of 2,3-BDO using bacterial species such as *Enterobacter* and *Klebsiella* is typically done owing to a fast growth in nutrient-poor media and the native 2,3-BDO-producing ability. For large-scale production, an inexpensive and abundant carbon source is one of the most important prerequisites. Therefore, previous efforts have used various biomass hydrolysates in the production 2,3-BDO. In one example, a research group tried to delete *pflB* (pyruvate formate-lyase) and *ptsG* (glucose permease of the phosphotransferase system) in *Enterobacter aerogenes* to increase their use of the mixed sugars in lignocellulosic hydrolysates. When a sugarcane bagasse hydrolysate was provided as a carbon source, the engineered *E. aerogenes* produced approximately 20 g/L 2,3-BDO (0.395 g 2,3-BDO/g carbon source) in 72 h [10]. Another engineered strain of *E. cloacae* produced 152 g/L 2,3-BDO within 44 h from lignocellulose-derived sugars [11]. In addition, other research group engineered *Klebsiella pneumonia* SDM using the strategy of random mutagenesis to produce 150 g/L 2,3-BDO by fed-batch fermentation by corn steep liquor [12]. Although these high performances of 2,3-BDO production are appealing, *Enterobacter* and *Klebsiella* species (except for *K. oxytoca*) are categorized as Risk Group 2 (RG2) pathogens by the World Health Organization [3]. Therefore, such strains might not be desirable for large-scale production of 2,3-BDO because of their pathogenic properties. Scientists tried to reduce the virulence of these pathogenic bacteria by disrupting a virulence-related *wabG* gene [13] and eliminating pathogenic factors (lipopolysaccharides, polysaccharide capsules, and fimbrial adhesins) [14]. Although their virulence factor was dramatically reduced, however, the concentration of 2,3-BDO was unfortunately decreased by 30% [13].

Instead of using a potentially pathogenic bacterial species, we suggest using *Saccharomyces cerevisiae*, a species listed as Generally Recognized As Safe (GRAS), to produce 2,3-BDO. It has been reported that engineered strains of *S. cerevisiae* rapidly and efficiently utilize various sugar sources, such as xylose [15], galactose [16], cellobiose [17] and hydrolysates from cassava [18], lignocellulose [19], and red algae [20]. Moreover, as industrial polyploid strains of *S. cerevisiae* exhibit high tolerance against alcohols, sugars, and harsh fermentation conditions [19, 21, 22], the 2,3-BDO production process would be improved if the polyploid *S. cerevisiae* is used. Nonetheless, genetic manipulation of polyploid *S. cerevisiae* has been limited, and thus, few attempts to metabolic engineering have been made for large-scale fermentation.

**Fig. 1:**
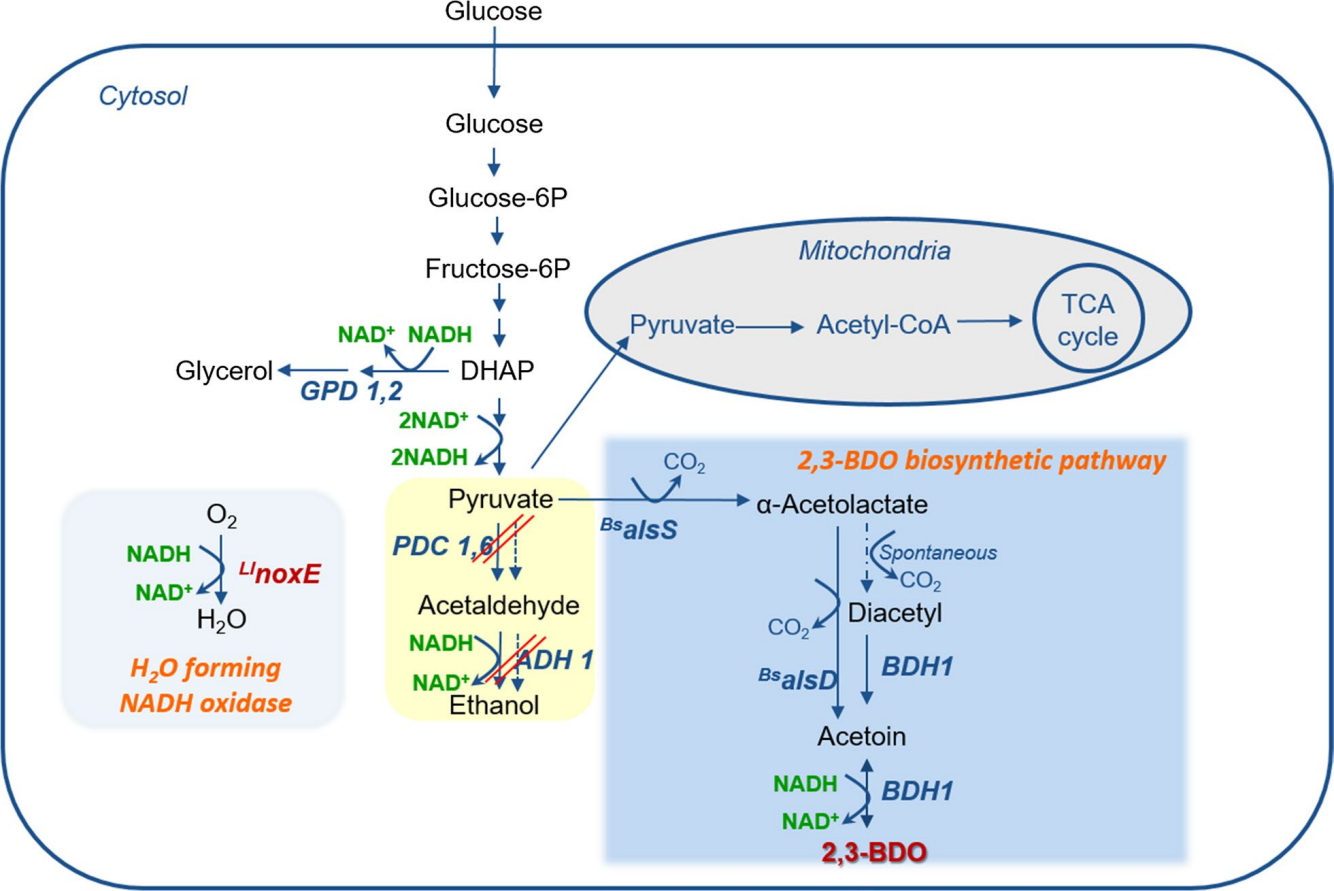
Schematic pathway for producing 2,3-BDO in industrial *S. cerevisiae*. *DHAP*: dihydroxyacetone phosphate; *GPD 1*,*2*: glycerol-3-phosphate dehydrogenase 1,2; ^*Ll*^*noxE*: NADH oxidase from *Lactococcus lactis*; *PDC 1,6*: pyruvate decarboxylase 1,6; *ADH 1*: alcohol dehydrogenase 1; ^*Bs*^*alsS*: α-acetolactate synthase from *Bacillus subtilis*; ^*Bs*^*alsD*: α-acetolactate decarboxylase from *B. subtilis*; *BDH1*: 2,3-butanediol dehydrogenase; 2,3-BDO: 2,3-butanediol. Dashed arrows indicate spontaneous steps in the cell and red diagonal double line means partially disruption

Normally, ethanol production dominates in *S. cerevisiae* sugar metabolism. This ethanol biosynthetic pathway needs to be metabolically replaced with a 2,3-BDO-producing pathway to produce 2,3-BDO efficiently (Fig. 1). As such, the previous research of our group made three perturbations to the laboratorial haploid *S. cerevisiae* genome to produce 2,3-BDO instead of ethanol from sugars. First, we deleted all three pyruvate decarboxylase isozymes (*PDC 1*, *5*, *6*), that convert pyruvate into acetaldehyde (the precursor of ethanol). Second, we introduced an enantiopure 2,3-BDO biosynthesis pathway including α-acetolactate synthase (*alsS*) and α-acetolactate decarboxylase (*alsD*) from *Bacillus subtilis*. Third, endogenous 2,3-butanediol dehydrogenase (*BDH1*) was over-expressed by a strong promoter of *TDH3* [23]. Although the resulting Pdc-deficient haploid *S. cerevisiae* strain produced 2,3-BDO as a major fermentation product without ethanol production, however, the engineered strain grew slowly on glucose medium [24, 25]. The impaired growth in the Pdc-deficient haploid strain resulted from the depletion, or limited synthesis of cytosolic acetyl-CoA which is produced from acetaldehyde via acetate [26]. To resolve the growth defect caused by the limited supply of cytosolic acetyl-CoA, the heterologous gene *PDC1* from *Candida tropicalis* was additionally expressed in the Pdc-deficient haploid *S. cerevisiae*, and the resulting strain produced 154.3 g/L 2,3-BDO. However, cell growth of pre-culture was still retarded without an ample supply of C_2_ compound [27]. In another study, the alcohol dehydrogenase (*ADH*) instead of *PDC* was deleted to address the limited production of Acetyl-CoA. However, an accumulation of acetaldehyde in this Adh-deficient *S. cerevisiae* inhibited cell growth, as well [28]. Thus, industrial-scale production of 2,3-BDO requires adjustment to these pdc- or adh-deficient *S. cerevisiae* strains.

We used an industrial polyploid *S. cerevisiae* obtained from the process of Korean liquor fermentation. It had been adapted to the severe environment and exhibited a rapid cell growth, a high sugar consumption rate, and a high tolerance to harsh conditions [19]. With these advantages for industrial large-scale production, in this study, the industrial *S. cerevisiae* was metabolically engineered to produce 2,3-BDO from glucose and cassava hydrolysates. To minimize production of ethanol as a dominant metabolite of *S. cerevisiae*, we attempted to disrupt simultaneously major isozymes of *PDC* and *ADH* gene without decreasing cell growth on glucose as a sole carbon source. In addition, we introduced the 2,3-BDO biosynthesis pathway consisting of an endogenous *BDH1* and *alsS* and *alsD* from *B. subtilis*. The *noxE* gene from *Lactococcus lactis* was coexpressed to maintain redox balance under reduced ethanol production. In addition to the genetic perturbations, fed-batch fermentations were optimized to improve 2,3-BDO productivity. Cultivating an engineered industrial polyploid *S. cerevisiae* strain capable of producing 2,3-BDO in combination with optimization of the fed-batch fermentation will allow economic and environmental-friendly production of 2,3-BDO from renewable biomass.

## Results

### Construction of a non-ethanologenic polyploid *S. cerevisiae*

#### Disruption of pyruvate decarboxylase (*PDC*)

**Fig. 2:**
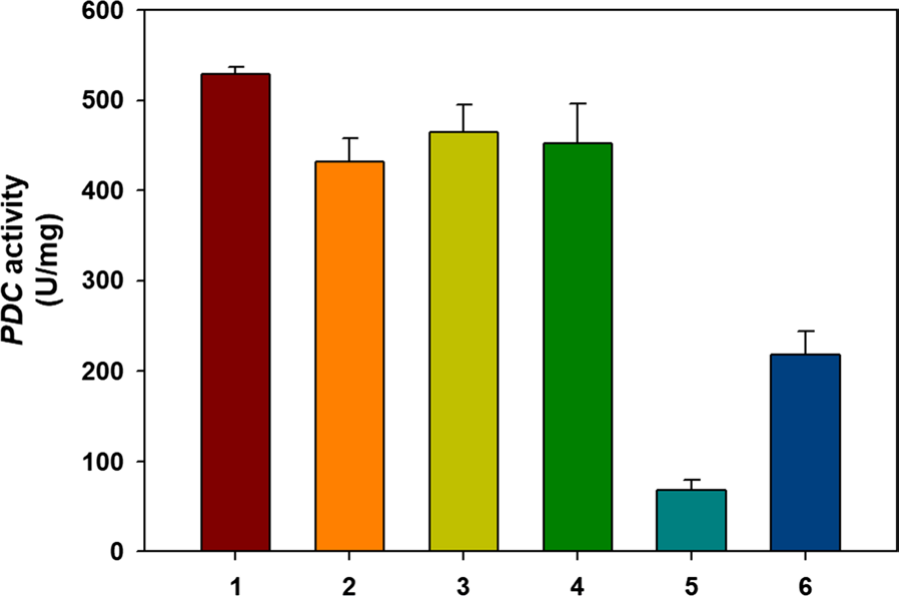
In vitro activity assay of endogenous *PDC* in engineered *S. cerevisiae* strains. Assays were replicated by four independent experiments. 1: 4-JHS200 (the control host strain); 2: 4-PDC1d (only *PDC1* disrupted); 3: 4-PDC5d (only *PDC5* disrupted); 4: 4-PDC6d (only *PDC6* disrupted); 5: 4-PDC16d (*PDC1* and *PDC6* disrupted); 6: 4-PDC56d (*PDC5* and *PDC6* disrupted)

The industrial polyploid *S. cerevisiae* (JHS200) was genetically manipulated using Cas9-based genome editing for the disruption of *PDC* as described previously [19]. A termination codon (TAA) was introduced in the middle of the open reading frames (ORFs) of each *PDC* isozymes (*PDC1*, *PDC5,* and *PDC6*). A quadruple auxotrophic strain (4-JHS200) [19] was used as a parental strain for the *PDC* disruption. First, *PDC1*, *PDC5*, and *PDC6* were individually disrupted in the 4-JHS200 strain, resulting in single *PDC*-disrupted strains (4-PDC1d, 4-PDC5d, and 4-PDC6d). The fermentation performance of the single *PDC*-disrupted strains was evaluated. Compared to the parental strain (4-JHS200), there was no difference in glucose consumption rate and production of ethanol and glycerol for three different *PDC*-disrupted strains (4-PDC1d, 4-PDC5d, and 4-PDC6d) (Additional file 1: Figure S1). According to a previous report, two leftover *PDC* genes provide enough Pdc activity for the cell growth even if one of the *PDC* isozymes was deleted [29]. Second, the 4-PDC1d and 4-PDC5d strains were subjected to additional disruption of *PDC*6 using the same method. These resulting strains of 4-PDC16d and 4-PDC56d (Table 1) were cultured in YP medium with 50 g/L of glucose to evaluate the effects of double disruption of *PDC* genes. As the 4-PDC16d strain has only *PDC*5 and the 4-PDC56d strain has only *PDC1*, we reasoned the partial disruption of *PDC* genes might maintain cell growth rate even without the supplement of C_2_-compounds. As previously reported, complete deficiency of Pdc activity in *S. cerevisiae* causes severe growth defects on glucose, and requires C_2_ compounds such as ethanol and acetic acid for growth [25, 27, 29]. As shown in the fermentation results (Additional file 1: Figure S1), the 4-PDC16d and 4-PDC56d strains produced ethanol and glycerol slightly less than the control strain (4-JHS200). Based on the amount of ethanol produced, we speculated that the 4-PDC16d strain might have a lower Pdc activity than the 4-PDC56d strain or the control strain. To confirm, we measured the Pdc activities of the *PDC*-disrupted strains. The double *PDC*-disrupted strains (4-PDC16d and 4-PDC56d) showed decreased Pdc activities as compared to the single *PDC*-disrupted strains (4-PDC1d, 4-PDC5d, and 4-PDC6d) (Fig. 2). The single *PDC*-disrupted strains maintained Pdc activity at a similar or slightly lower level compared with the control strain (4-JHS200), and the 4-PDC16d strain exhibited the lowest Pdc activity (Fig. 2). Thus, the 4-PDC16d strain with the attenuated Pdc activity was viewed as beneficial as a platform strain to produce 2,3-BDO instead of ethanol from pyruvate.

**Table 1:** Strains and plasmids used in this study

Strains	Genotype	Reference
4-JHS200	JHS200, *ura3Δ trp1Δ his3Δ leu2Δ*	[19]
4-PDC1d	4-JHS200, *pdc1Δ*	In this study
4-PDC5d	4-JHS200, *pdc5Δ*	In this study
4-PDC6d	4-JHS200, *pdc6Δ*	In this study
4-PDC16d	4-JHS200, *pdc1Δ pdc6Δ*	In this study
4-PDC56d	4-JHS200, *pdc5Δ pdc6Δ*	In this study
YG01	4-PDC16d, *Δadh1::TDH3*_prom_-*Llnox*-*CYC1*_term_	In this study
YG01_SDB	YG01, p413_SDB	In this study
YG01_SDBN	YG01, p413_SDB, p426TDH3_Llnox	In this study
YG02	4-PDC56d, *Δadh1::TDH3*_prom_-*Llnox*-*CYC1*_term_	In this study
YG02_SDB	YG02, p413_SDB	In this study

Plasmids	Description	Reference
Cas9_Aur	p414-TEF1p-Cas9-CYC1t, modified Cas9 expression plasmid	[19]
pRS42H	Backbone plasmid for constructing guideRNA plasmids	[40]
gRNA_dPDC1	pRS42H harboring *PDC*1 disruption gRNA cassette	In this study
gRNA_dPDC5	pRS42H harboring *PDC*5 disruption gRNA cassette	In this study
gRNA_dPDC6	pRS42H harboring *PDC*6 disruption gRNA cassette	In this study
gRNA_dADH1	pRS42H harboring *ADH*1 disruption gRNA cassette	In this study
p413_SDB	2,3-BDO biosynthesis pathway (plasmid p413 harboring *alsS*, *alsD*, *BDH*1)	[28]
p426TDH3_Llnox	p426TDH3 harboring *noxE* gene from *L. lactis* and template of NADH oxidase expression cassette *TDH3*p-*Llnox*-*CYC1*t	[32]

#### Deletion of alcohol dehydrogenase (*ADH*) with chromosomal expression of NADH oxidase (*noxE*)

In addition to *PDC* disruption, the expression levels of alcohol dehydrogenases (*ADH*), which convert acetaldehyde to ethanol (Fig. 1), can be modulated to reduce ethanol production. It has been known that there are five isozymes (*ADH1*–*ADH5*) of alcohol dehydrogenase in *S. cerevisiae*. However, similar to the Pdc-deficient strain, the Adh-deficient strain also had fatal features, such as an intracellular redox imbalance and deficient cell growth caused by accumulation of acetaldehyde [30, 31]. Therefore, *ADH1* known as the major active enzyme out of the five isozymes would be deleted for reducing ethanol production [30]. Since deletion of the NADH-dependent *ADH1* gene might cause cofactor imbalance, the heterologous water-forming NADH oxidase (*noxE*) from *L. lactis* was simultaneously introduced in the middle of *ADH1* chromosome. An expression cassette (including *TDH3* promoter and *CYC1* terminator) of NADH oxidase was amplified from the plasmid of p426TDH_Llnox (Table 1). This expression cassette of NADH oxidase (*noxE*) was introduced into the middle of the *ADH1* chromosome in the 4-PDC16d and 4-PDC56d strains, resulting in the deletion of *ADH1* with expression of *noxE*. The resulting strains were named as YG01 (4-PDC16dADH1d) and YG02 (4-PDC56dADH1d), respectively. The YG01 strain has intact *PDC5* and deleted *ADH1* by the chromosomally expression of *noxE*, and the YG02 strain has intact *PDC1* and deficient *ADH1* by *noxE* expression.

These two resulting strains were cultured in YP medium with 40 g/L glucose. Both YG01 and YG02 strains did not grow well, but produced a small amount of ethanol (< 2 g/L) (Additional file 1: Figures S2, S3). If the YG01 and YG02 strains lost their Adh activity by expression of *noxE* in the middle of the *ADH1* chromosome, acetaldehyde would not be converted to ethanol and accumulated in the cell (Fig. 1). In fact, we detected that 55 mg/L of acetaldehyde accumulated in the YG01 and YG02 cells, which was a 100-fold higher than that in the control strain (4-PDC16d) (data not shown). The accumulated acetaldehyde could not be converted to ethanol due to the weakened Adh activity in the YG01 and YG02 strains.

### Introduction of a 2,3-BDO pathway to the non-ethanologenic yeast strains

The next step was to introduce a 2,3-BDO biosynthetic pathway consisting of *alsS* and *alsD* from *B. subtilis* and over-expression of endogenous *BDH1* in the non-ethanologenic strains, YG01 and YG02. Specifically, the plasmid p413_SDB containing *alsS*, *alsD*, and *BDH1* under strong promoters was introduced into the YG01 and YG02 strains. Thus, carbon fluxes from pyruvate were redirected into α-acetolactate using α-acetolactate synthase encoded by *alsS*. Then, α-acetolactate was converted into acetoin via α-acetolactate decarboxylase encoded by *alsD*. The butanediol dehydrogenase enzyme encoded by *BDH1* converted acetoin into 2,3-BDO. The resulting strains were named as YG01_SDB and YG02_SDB (Table 1).

**Fig. 3:**
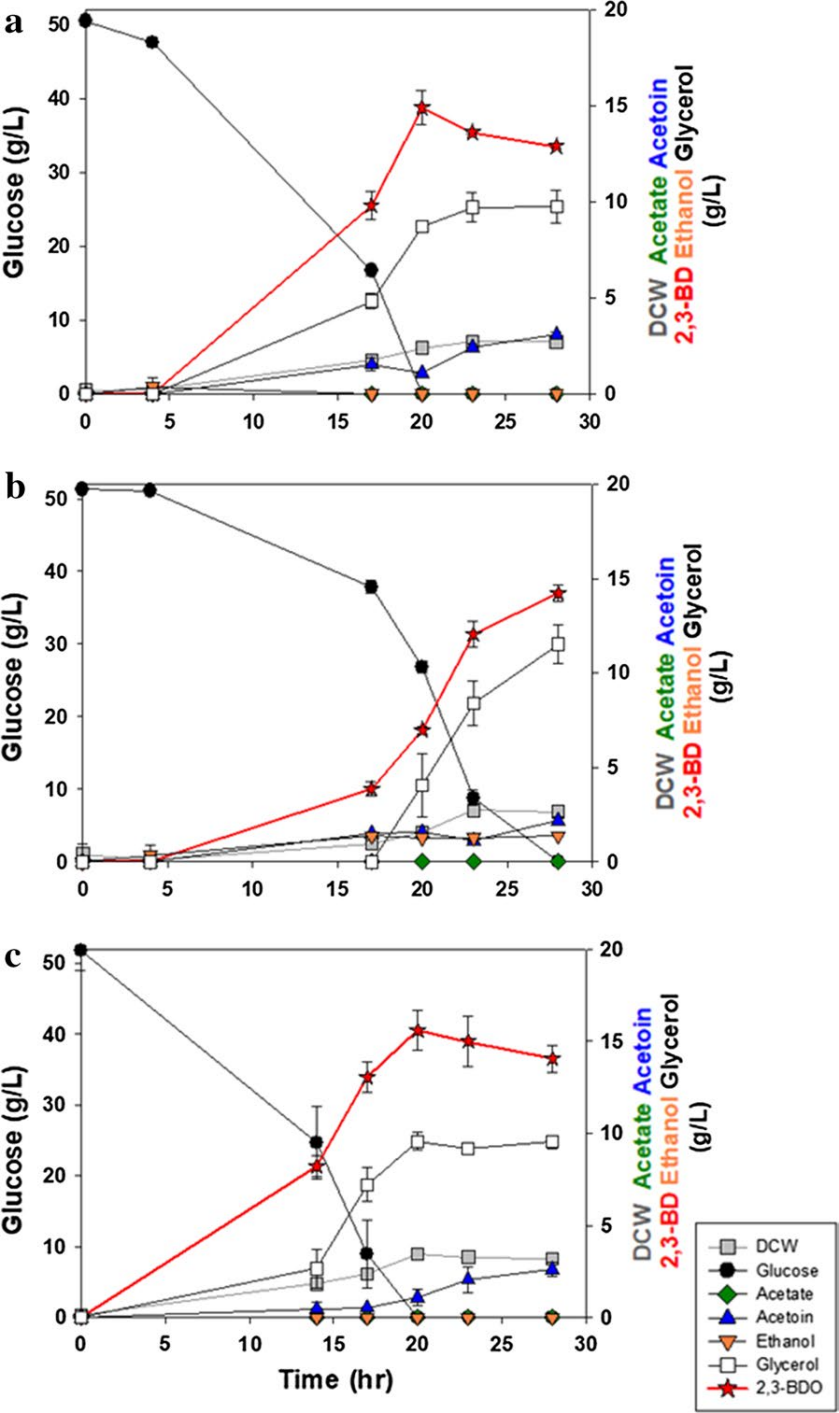
Flask fermentation of **a** YG01_SDB, **b** YG02_SDB, and **c** YG01_SDBN strains with 50 g/L glucose in YP medium. Error bars indicate standard deviations of three independent experiments

To evaluate fermentation performance changes after introducing the 2,3-BDO biosynthetic pathway, the YG01_SDB and YG02_SDB strains were cultured in a flask containing YP medium with 50 g/L of glucose. As shown in Fig. 3, the YG01_SDB strain was found to be more suitable for the production of 2,3-BDO than the YG02_SDB strain. The glucose consumption rate of the YG01_SDB strain was faster than that of the YG02_SDB strain. As a result, the cell concentration of the YG01_SDB strain reached a maximum faster than the YG02_SDB strain (Fig. 3a, b). Moreover, the YG02_SDB produced a small amount of ethanol (< 2 g/L) during the fermentation. Therefore, the YG02_SDB strain was excluded for further experiments. As summarized in Table 2, the YG01_SDB strain produced 14.9 g/L of 2,3-BDO from 50 g/L glucose within 20 h. Although a substantial amount (9.56 g/L) of glycerol accumulated, the YG01_SDB yielded 0.295 g 2,3-BDO/g glucose at a rate of 0.745 g/L h in a flask fermentation. After 20 h, the 2,3-BDO product was gradually converted into acetoin. Once glucose was depleted, it was likely the lack of NADH affected the cell growth. As such, acetoin accumulated from the oxidation of 2,3-BDO into acetoin for regenerating NADH. As the total amount of 2,3-BDO and acetoin remained unchanged, we speculated that acetoin was not metabolized further for respiratory growth [28].

**Table 2:** Summary of the flask culture with the engineered industrial yeast strains

Strains	Products (g/L)					Yield of 2,3-BDO (g 2,3-BDO/g glucose)	Productivity of 2,3-BDO (g/L h)
	DCW	2,3-BDO	Glycerol	Acetoin	Ethanol		
YG01_SDB	2.72	14.9	9.56	3.11	N/D	0.295	0.745
YG02_SDB	2.65	14.2	9.97	2.20	1.37	0.277	0.508
YG01_SDBN	3.19	15.6	9.78	2.62	N/D	0.301	0.781

*N/D* not detected

### Relieve of cellular redox imbalance by over-expressed NADH oxidase

Typically, alcohol dehydrogenase converts two molecules of acetaldehyde into two moles of ethanol with consumption of two moles of NADH (Fig. 1). Therefore, deleting the *ADH1* gene caused an accumulation of NADH in the cytosol of the non-ethanologenic strain (YG01). The surplus NADH produced during 2,3-BDO biosynthesis usually could be reoxidized into NAD^+^ via the glycerol overproduced [28]. However, the accumulation of glycerol obstructs the efficient production of 2,3-BDO as substantial amounts of a carbon source can be wastefully incorporated into glycerol. In addition, downstream processing will be complicated due to the similar chemical properties between glycerol and 2,3-BDO. To reduce the accumulation of glycerol, surplus NADH can be efficiently oxidized into NAD^+^ using a water-forming NADH oxidase from *L. lactis* [32]. When the *ADH1* gene was deleted, an expression cassette of *noxE* was inserted into the *ADH1* gene site in the chromosome of the YG01_SDB strain. However, the chromosomal single copy-number-based expression was not sufficient to compete for NADH with *GPD* (glycerol-3-phosphate dehydrogenase), resulting in accumulation of glycerol during 2,3-BDO biosynthesis (Fig. 3a). Therefore, a multi-copy-number plasmid (p426TDH3_Llnox) was introduced into the YG01_SDB strain for enhancement of NADH oxidase activity. The resulting strain (YG01_SDBN) indicated a lower glycerol yield than the parental strain (YG01_SDB) (Fig. 3b, c). As expected, the NADH oxidase expression levels were inversely proportional to the glycerol accumulation levels, and proportional to 2,3-BDO production (Table 2). While the YG01_SDBN produced glycerol, we reasoned it is because of limited supply of oxygen in a flask fermentation.

**Fig. 4:**
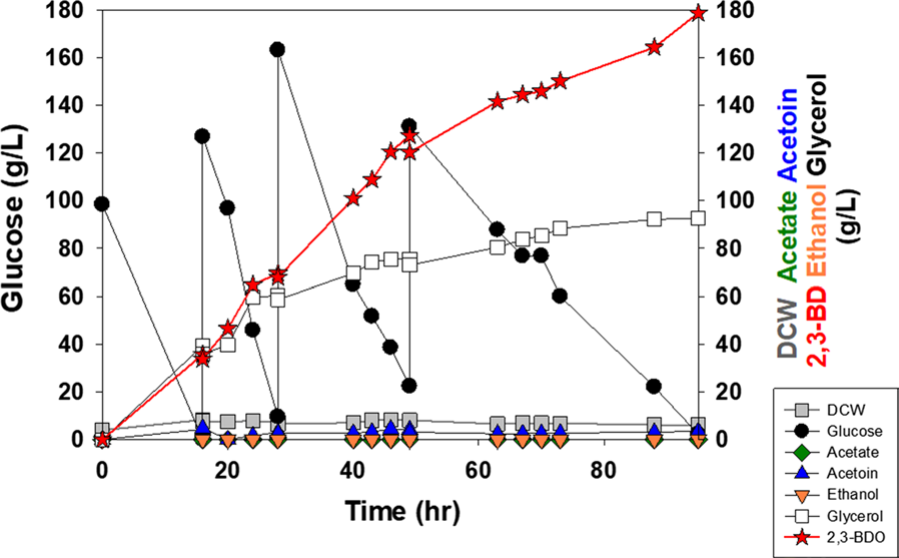
Fed-batch fermentation of YG01_SDBN strain in a fermentor with YP medium

To maintain the oxygen level, a fed-batch fermentation was conducted in a 1 L fermentor with the YG01_SDBN (Fig. 4). After the initially added glucose (100 g/L) was depleted, 800 g/L glucose-concentrated solution was intermittently fed when glucose concentration decreased to 20 g/L or less during the fermentation. Aeration conditions were controlled at 400 rpm and a flow rate of 2 vvm. The dissolved oxygen level in the medium was kept below 2.0%. The fed-batch fermentation showed two phases of 2,3-BDO production (Fig. 4). For the first 49 h of fermentation, the YG01_SDBN strain could produce high concentrations of 2,3-BDO (129 g/L) with a high rate of 2.64 g/L h, which is comparable to the fermentation performances of bacteria-based processes. After 49 h of fermentation, 2,3-BDO production increased to 178 g/L, at the expense of overall productivity, which declined from 2.64 to 1.88 g/L h, probably because of high osmotic pressure caused by high concentrations of 2,3-BDO (Table 3). No ethanol production was observed in the fed-batch fermentation. With these fermentation results, the metabolically engineered industrial polyploid *S. cerevisia*e strain (YG01_SDBN) constructed in this work has successfully demonstrated the possibility of producing high concentration of 2,3-BDO with high productivity.

**Table 3:** Summary of fermentations with the YG01_SDBN strain in a fermentor

Fed-batch fermentation with YP medium	Up to 49 h	Overall					
Concentration of 2,3-BDO (g/L)	129	178					
Yield of 2,3-BDO (g 2,3-BDO/g glucose)	0.313	0.335					
Productivity of 2,3-BDO (g/L h)	2.64	1.88					

Fermentation with cassava hydrolysates	Products (g/L)					Yield of 2,3-BDO (g 2,3-BDO/g glucose)	Productivity of 2,3-BDO (g/L h)
	DCW	2,3-BDO	Glycerol	Acetoin	Ethanol		
Batch	9.55	81.2	51.5	1.17	N/D	0.305	1.89
Fed-batch	8.45	132	69.4	1.19	N/D	0.324	1.92

*ND* not detected

### Production of 2,3-BDO from a cassava hydrolysate

**Fig. 5:**
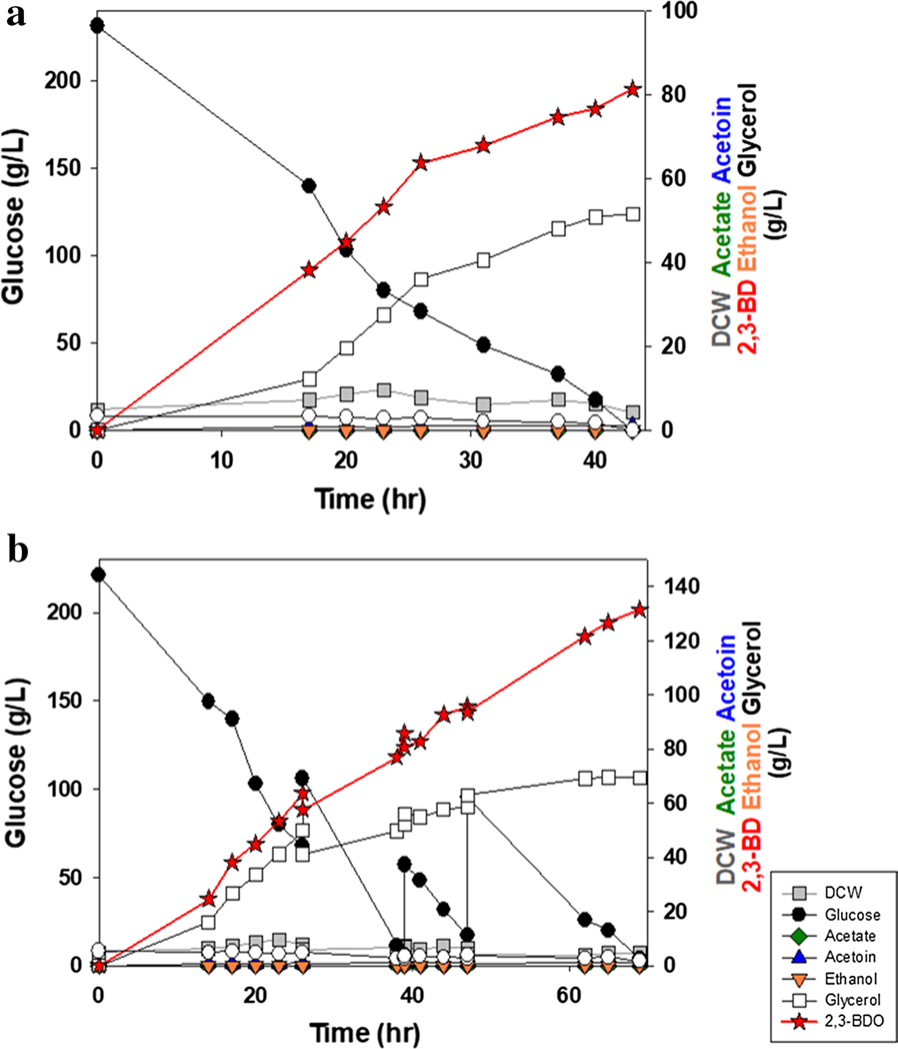
Fermentation of YG01_SDBN strain in a fermentor with a cassava hydrolysate. **a** Batch and **b** fed-batch fermentation

To be competitive with chemical synthesis of 2,3-BDO, a microbial fermentation process should produce 2,3-BDO efficiently from inexpensive substrates. As shown in previous studies, the same parental strain (JHS200), such as the engineered polyploid *S. cerevisiae* strain used in this study, has outstanding examples of fermenting a cellulosic hydrolysate containing substantial amounts of fermentation inhibitors [19]. Therefore, we evaluated the production of 2,3-BDO by the YG01_SDBN strain with a cassava hydrolysate. The batch culture of a cassava hydrolysate was carried out using the YG01_SDBN strain in a 1 L scale fermentor (Fig. 5a). The cassava hydrolysates contained 266 g/L of glucose as a carbon source, and 10 g/L of yeast extract and 20 g/L of peptone were added as a nitrogen source. During 43 h of the batch fermentation, 81.2 g/L 2,3-BDO was produced with a productivity of 1.89 g/L h and a yield of 0.305 g 2,3-BDO/g glucose (Table 3). These results are comparable to rates of bacterial fermentations of hydrolysates [33].

A fed-batch fermentation using the cassava hydrolysate was also performed under the same conditions of aeration and feeding strategies as done in the YP medium-based fermentation. The fed-batch fermentation of the cassava hydrolysate with the YG01_SDBN strain produced 132 g/L of 2,3-BDO within 69 h with a productivity of 1.92 g/L h (Fig. 5b). While the production of 2,3-BDO continued to increase at a constant rate, the amount of glycerol reduced, so the 2,3-BDO yield (0.324 g 2,3-BDO/g glucose) was greater than the yield of the batch fermentation (0.305 g 2,3-BDO/g glucose). Although the cassava hydrolysate contained a high concentration of glucose, the YG01_SDBN strain was able to produce 2,3-BDO with a better yield and productivity from the cassava hydrolysate than from YP medium with glucose. Cassava has been known as an accessible substrate in tropical regions such as Southeast Asia and Africa, and its hydrolysate could be a good biomass [34]. With this aspect, this constructed industrial polyploid *S. cerevisiae* strain can be employed to produce 2,3-BDO with a high productivity using inexpensive substrates such as a cassava hydrolysate.

## Discussion

This study aimed that the microbial production of 2,3-BDO would be improved by an industrial polyploid *S. cerevisiae* with high titer and productivity. As the industrial yeast strain has fast growth rate, sugar metabolism, and resistance to fermentation inhibitors, it is suitable for the large-scale fermentation with a biomass hydrolysate in industrial fields [19]. Furthermore, if genetic modification is successfully carried out in an industrial polyploid *S. cerevisiae* without some problems shown in previous studies, the engineered polyploid strain can produce high titer of 2,3-BDO with high productivity as much as engineered bacteria. Although some bacteria species are native producers of 2,3-BDO, most of them are classified into the Risk Group 2, which contains pathogenic organisms unsuitable for the large-scale fermentation. Possibility of fatal infection by bacteriophage should also be resolved in bacterial production of 2,3-BDO [35]. As such, the improved 2,3-BDO biosynthesis of the industrial polyploid *S. cerevisiae* could be feasible in the production of 2,3-BDO from renewable biomass. Though a polyploid *S. cerevisiae* has various advantages for large-scale fermentation process, its genetic manipulation has been limited [36]. The CRISPR-Cas9 genome-editing technology could be applicable to manipulating the genome of industrial polyploid yeast in this study.

We hypothesized that an expression level of the pyruvate decarboxylase (*PDC*) and alcohol dehydrogenase (*ADH*) genes could be modulated to construct a non-ethanologenic strain capable of growing on glucose as a sole carbon source without supplementation of a C_2_-compound. Using an industrial polyploid *S. cerevisiae* as a parental strain, partial disruption of *PDC* isozymes was combined with partially deleted *ADH* isozymes to minimize ethanol production from glucose as a sole carbon source without growth inhibition. Among the three isozymes (*PDC1*, *5*, 6) of pyruvate decarboxylase, the combination of disrupted *PDC1* and *PDC6* was exhibited the lowest activity of *PDC* (Fig. 2). This partial disruption of *PDC* isozymes had solved the problems of C_2_-auxotrophy and growth inhibition, which typically occurred in Pdc-deficient laboratorial haploid yeast strains [24, 27]. There has been reported chromosomal instability, however, caused by incomplete deletion of genes in polyploid *S. cerevisiae*. To confirm the stability of genetic modifications, we investigated the genome sequence by DNA sequencing. As shown in Additional file 1: Figure S4, genetic manipulation of inserting the stop codon (TAA) has been maintained from the first transformants (left side) to the 60th generations strain (right side).

**Fig. 6:**
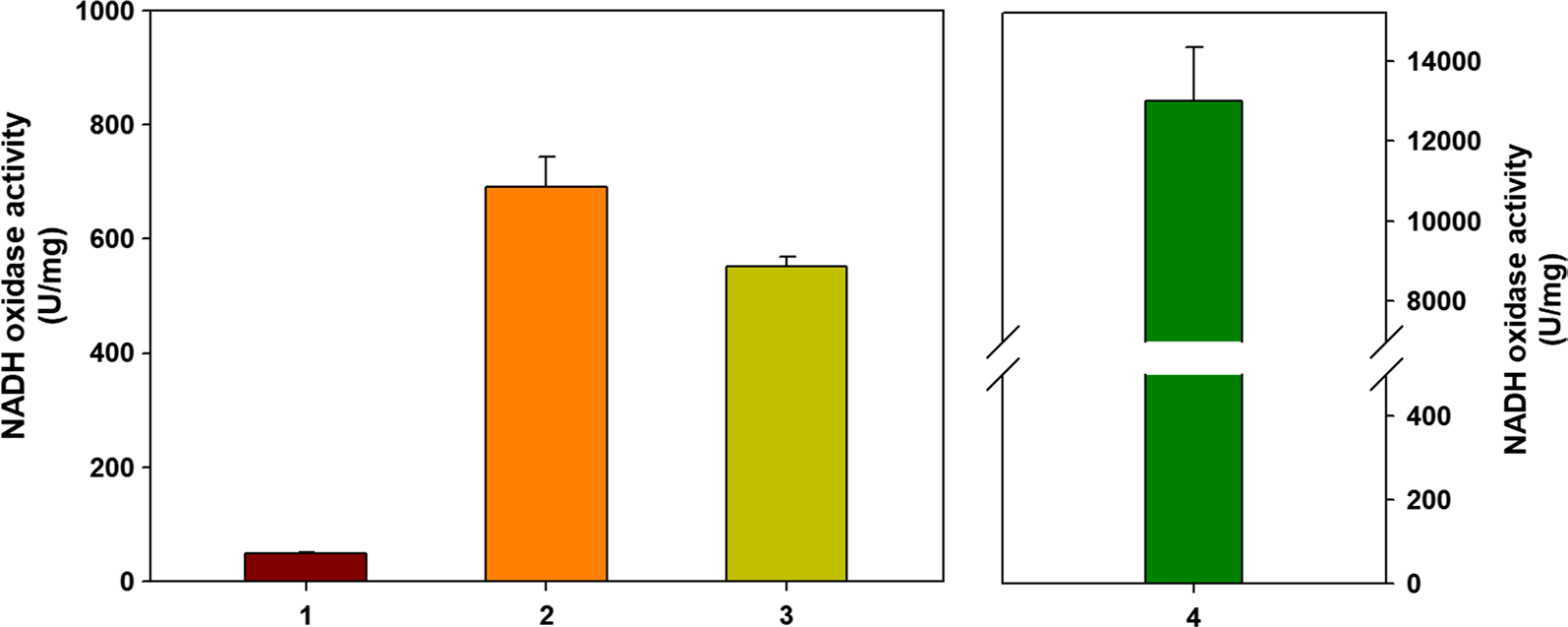
In vitro activity assay of heterologous NADH oxidase expressed in engineered *S. cerevisiae* strains. Assays were replicated by four independent experiments. 1: 4-JHS200 (the parental strain); 2: YG01_SDB (partially disrupted *PDC1*, *PDC6,* and *ADH1* genes with the chromosomal expression of *noxE* gene and introduced 2,3-BDO pathway); 3: YG02_SDB (partially disrupted *PDC5*, *PDC6,* and *ADH1* genes with the chromosomal expression of *noxE* gene and introduced 2,3-BDO pathway); 4: YG01_SDBN (partially disrupted *PDC1*, *PDC6,* and *ADH1* genes with the chromosomal expression of *noxE* gene, introduced 2,3-BDO pathway and expressed with the plasmid of p426TDH3_Llnox)

Furthermore, by eliminating only the Adh1 activity among the six isozymes of *ADH*, ethanol biosynthesis could be minimized without a severe metabolic burden. As a result, a non-ethanologenic polyploid *S. cerevisiae* (YG01) could not produce ethanol at all and was barely able to grow. Partial disruption of *PDC* and *ADH* isozymes blocked the metabolic pathway from pyruvate to ethanol, resulting in the accumulation of acetaldehyde (55 mg/L) in the cells, which prohibited cell growth (Additional file 1: Figures S2, S3). The introduction of the 2,3-BDO biosynthetic pathway allowed the massive shift of carbon flux from pyruvate to 2,3-BDO. Since the NADH oxidase had been introduced at the *ADH1* chromosome site in the process of *ADH1* disruption, the level of NADH expression could be an indicator measured to confirm the disruption of *ADH1* and chromosomal expression of *noxE*. To determine the expression level of the *noxE* gene, the NADH oxidase activity was measured for the YG01_SDB and YG02_SDB. As mentioned above, the YG01 and YG02 strains struggled to grow, so NADH oxidase activity was measured in the YG01_SDB and YG02_SDB strains. These strains were genetically identical to YG01 and YG02 except for additional introduction of the 2,3-BDO pathway. The chromosomally *noxE* expressed strains (YG01_SDB and YG02_SDB) showed higher activity than the control strain (4-JHS200), indicating the confirmation of the successfully introduced heterologous *noxE* gene (Fig. 6). In conclusion, the non-ethanologenic polyploid *S. cerevisiae* strains (YG01 and YG02), which were constructed by partially disrupting *PDC* and *ADH* by introduction of the heterologous *noxE* gene, are a promising platform to biosynthesize various products from pyruvate.

Based on the glycerol production yield of YG01_SDBN and YG01_SDB strain, over-expression of NADH oxidase relieved the redox imbalance caused by blocking the ethanol biosynthetic pathway which oxidized cellular NADH. Production of glycerol which oxidizes excessive NADH into NAD^+^ in the cell, however, inevitably occurred in the process of 2,3-BDO biosynthesis. Thus, a water-forming NADH oxidase was introduced to alleviate the intracellular imbalance in the NADH/NAD^+^ ratio and to reduce the glycerol accumulation [27]. Under the optimized fermentation conditions, the resulting engineered industrial strain (YG01_SDBN) produced 178 g/L 2,3-BDO with a maximum productivity of 2.64 g/L h in a fed-batch fermentation using glucose-based YP medium.

For a cassava hydrolysate, the YG01_SDBN strain could also produce 2,3-BDO while maintaining its advantageous properties such as tolerance to sugars and fermentation inhibitors at high concentrations, and rapid sugar consumption rates. This strain under fed-batch conditions using a cassava hydrolysate produced 132 g/L of 2,3-BDO with a yield of 0.324 g 2,3-BDO/g glucose. These results demonstrate that an engineered industrial polyploid *S. cerevisiae* might serve as a technical platform to produce valuable chemicals from inexpensive hydrolysates of renewable biomass.

## Conclusions

In this study, we introduced the 2,3-BDO biosynthesis system (*alsS*, *alsD* from *B. subtilis* with over-expressed endogenous *BDH1*) into an industrial polyploid *S. cerevisiae* and produced 2,3-BDO very efficiently. Unlike previous studies [24, 27, 28], the combination of disrupted *PDC1/PDC6* and *ADH1* rerouted carbon fluxes into the 2,3-BDO biosynthesis pathway instead of into ethanol production and avoided any severe growth defects. We successfully developed an engineered industrial polyploid *S. cerevisiae* strain (YG01_SDB and YG02_SDB) able to overcome C_2_-dependent growth and display various advantages among industrial strains when producing 2,3-BDO. In addition, the concentration of 2,3-BDO (178 g/L) by the engineered polyploid *S. cerevisiae* strain (YG01_SDBN) is one of the highest titer reported for microbial production of 2,3-BDO. While microbial production of 2,3-BDO has been studied in various species of microorganism and various ways [37], attempts in an industrial polyploid *S. cerevisiae* are novel [38]. Besides, the results of cassava hydrolysate fermentation using the engineered polyploid *S. cerevisiae* are not inferior even compared with those of bacteria including *K. oxytoca* [39]. We expect that an engineered polyploid *S. cerevisiae* could be a powerful platform strain for production of biofuels and biochemicals with environmental and economic benefits.

## Methods

### Strains and culture media

*Escherichia coli* Top 10 (Invitrogen, Carlsbad, CA, USA) was used for plasmid construction and gene manipulation. We grew the *E. coli* transformants in Lysogeny Broth (LB) medium with 100 μg/mL ampicillin. The industrial *S. cerevisiae* strain used in this study was found to be polyploid. The engineered polyploid 4-JHS200 was described in a previous study [19]. We cultivated the yeast strains in YP (10 g/L yeast extract and 20 g/L peptone) medium with 0.5 μg/mL aureobasidin A (Takara, Shiga, Japan) or 300 μg/mL hygromycin B (Sigma-Aldrich, St. Louis, MO, USA). Some of the engineered industrial strains were selected on YNB medium (6.7 g/L yeast nitrogen base, proper nucleotides and amino acids) with 0.5 μg/mL aureobasidin A and 300 μg/mL hygromycin B as selection markers.

### Construction of plasmids

Table 1 summarizes the plasmids used in this study. Primers used to construct gBlock and repair DNA are presented in Additional file 1: Table S1. The Cas9 expression plasmid named Cas9_Aur was used [19]. Customized guide RNA-expressing plasmids were constructed as follows. First, three guide RNA-expressing cassettes with different sequences targeting *PDC1*, *PDC5,* and *PDC6* were synthesized to have blunt ends for *Sac*I and *Kpn*I digestion. The AccuGeneBlock service (Bioneer Co., Korea) synthesized these guide RNA cassettes (Additional file 1: Table S2). Second, the synthesized guide RNA cassettes and plasmid pRS42H [40] were digested using *Sac*I and *Kpn*I, and ligated to each other. Third, we obtained the guide RNA-expressing plasmids, gRNA_dPDC1, gRNA_dPDC5, and gRNA_dPDC6 for disruption of *PDC1*, *PDC5,* and *PDC6*, respectively. The guide RNA-expressing plasmids for the disruption of *ADH*1 (gRNA_dADH1) [41] and the plasmid p426TDH3_Llnox were described in a previous study [32]. The constructed 2,3-BDO pathway was introduced via plasmid p413_SDB, which was kindly donated by Prof. Ji-Sook, Hahn [28]. This plasmid p413_SDB consists of a heterogeneous *alsS* and *alsD* from *B. subtilis* and an over-expressed innate gene *BDH1* under the control of strong constitutive promoters (P_*TDH3*_, P_*TEF1*_, and P_*TPI1*_, respectively), and had different terminators [28].

### Construction of metabolically engineered *S. cerevisiae* by transformation

For the construction of *PDC*-disrupted strains, 4-JHS200 harboring Cas9-Aur (Cas9-expression plasmid) was transformed with guide RNA plasmids and repair DNA fragments amplified by PCR. To introduce the 2,3-BDO biosynthetic pathway, plasmid p413_SDB was transformed into the YG01 and YG02 strains using the Spheroplast Transformation Kit (BIO 101, Vista, CA, USA). To select the transformants, *S. cerevisiae* strains were cultivated aerobically at 30 °C in YNB medium (6.7 g/L Yeast Nitrogen Base, appropriate nucleotides, and amino acids) with 20 g/L glucose.

### Activity assay of NADH oxidase and pyruvate decarboxylase

To measure NADH oxidase activity in a cell, a crude extract was prepared by growing and harvesting the mid-exponential phase cells (up to 1 × 10^8^ to 10^9^) grown in YNB medium-containing 20 g/L glucose, followed by two washes of distilled water. After adding the protease inhibitor (Roche, Basel, Switzerland), the harvested cells were lysed using the yeast protein extraction reagent (Thermo Scientific, Waltham, MA, USA). Cells were centrifuged at 7000*g* and 4 °C for 20 min, and the supernatant was collected and tested for NADH oxidase activity within 3 h. The reaction mixture contained 50 mM potassium phosphate buffer (pH 7.0), 0.4 mM NADH, and 0.3 mM EDTA [42]. Enzyme activity was defined as the degree of absorbance reduction at 340 nm. One unit is defined as the enzyme amount needed to oxidize 1 μmol NADH under the conditions mentioned above for 3 min.

To measure the Pdc activity in the cells, we prepared a crude extract using the methods already described. The Pdc activity was assayed at 30 °C with a reaction mixture containing 40 mM imidazole hydrochloride buffer (pH 6.5), 5 mM MgCl_2_, 0.2 mM TPP, 17 U of alcohol dehydrogenase from *S. cerevisiae*, 0.4 mM NADH, and 50 mM pyruvate [43]. We initiated the reactions by adding pyruvate and measured the change in absorbance at 340 nm. One unit of activity was defined as the amount of enzyme oxidizing 1 μmol NADH per minute in the corresponding reaction conditions. The protein concentration of crude extracts was also determined by the Bradford method [44].

### Culture conditions

The engineered yeast strains were pre-cultured in 5 mL of YP medium or YNB medium-containing 20 g/L glucose at 30 °C and 250 rpm for 24 h. After growth, cells were harvested and transferred to 100 mL of YNB medium-containing 20 g/L glucose in a baffled flask at 30 °C and 250 rpm for 24 h. These cells at mid-exponential growth state (OD_600_ < 3) were harvested for use as an inoculum. Main batch cultures were carried out under microaerobic conditions in 250 mL Erlenmeyer flasks at 30 °C and 150 rpm. Fed-batch fermentations were done using a 1 L bench-top fermentor (Fermentec, Cheongju, Korea) filled with 500 mL YP medium-containing initial 100 g/L glucose and held at 30 °C. The pH was targeted to 5.5 by the intermittent addition of 5 N NaOH solution. An O_2_ sensor (Mettler Toledo, Greifensee, Switzerland) was used to measure the dissolved oxygen (DO). Fed-batch cultivation was done using a fermentor operated at 400 rpm with an air flow rate of 2 vvm. During the fed-batch fermentation, DO levels were kept below 2.0%. Cells prepared from the flask culture were used to inoculate the fed-batch culture at an initial concentration of 5.0 g_DCW_/L. When glucose concentration fell below 20 g/L, glucose-concentrated solution (800 g/L) was added into the culture broth.

For the hydrolysate fermentations, 10 g/L yeast extract and 20 g/L peptone were added into the cassava hydrolysates from Changhae Ethanol Co., Ltd. Other fermentation conditions are the same as the above description.

### Preparation of cassava hydrolysates

Enzymatic hydrolysis can be used to obtain 100 g glucose from 126 g of the cassava chips. Using this information, cassava hydrolysates were prepared from cassava chips (starch contents equal to approximately 72% dry weight basis) imported from Vietnam. Cassava chips were ground using a hammer mill and passed through a 1 mm screen. For liquefaction, 0.7 g/kg dry matter of commercial α-amylase (Termamyl SC, Novozymes, Bagsvaerd, Denmark) was added, and the mash was heated to 100 °C and liquefied for 90 min. After the liquefying steps were completed, the resulting mash was cooled to a saccharification temperature (50 °C), and Spirizyme Fuel (Novozymes, Bagsvaerd, Denmark) was added at 0.5 g/kg dry matter. This saccharification step was aseptically performed for 24 h [45]. The chemically saccharified cassava hydrolysates were used for fermentation experiments.

### Determination of dry cell weight and metabolites

We measured the cell growth based on optical density using a spectrophotometer (OPTIZEN POP; Mecasys, Daejeon, Korea) set at 600 nm (OD_600_). Dry cell weight (DCW) was estimated using a conversion factor of 0.50 g_dry cell_/L/OD_600_. Acetate, acetoin, ethanol, glucose, glycerol, and 2,3-BDO were measured using a high-performance liquid chromatography (1260 Infinity, Agilent, CO, USA) equipped with a BioRad Aminex HPX-87H column (300 mm 7.8 mm, 5 µm; Bio-Rad, Hercules, CA, USA) and a 5 mM H_2_SO_4_ mobile phase set at a flow rate of 0.6 mL/min. Metabolites were quantified using a refractive index (RI) detector.

## Supplementary information

**Additional file 1.** Additional tables and figures.

## Data Availability

Data made available to all interested researchers upon request.
