# Supplementary material for: Production of 2,3-butanediol from glucose and cassava hydrolysates by metabolically engineered industrial polyploid Saccharomyces cerevisiae
Source: Biotechnol Biofuels. 2019 Aug 29;12:204. doi: 10.1186/s13068-019-1545-1 (PMC6714309; doi:10.1186/s13068-019-1545-1)

Table S1. Primers used in this study.

Primer name	Sequences
**F_PDC1(stop)_repair**	gctaagaacccagttatcttggctgatgcttgttgttccagacacgacgtcaagtaagaa
**R_PDC1(stop)_repair**	gaattgagtcaagtcaatcaacttcttagtttcttacttgacgtcgtgtctggaacaaca
**F_PDC5(stop)_repair**	atttatttgaaagattgagccaagtcaactgtaacaccgtcttcggtttgccagtaaact
**R_PDC5(stop)_repair**	cataaagcttatccaaaagagacaagttaaagtttactggcaaaccgaagacggtgttac
**F_PDC6(stop)_repair**	ggtgtttatgtgggaacgctgtccaaaccagacgtgaaacaggccgttgagtcgtaagat
**R_PDC6(stop)_repair**	agagagcaaagcaccgaccgaaaggatcaaatcttacgactcaacggcctgtttcacgtc
**F1_SacI_gBlock**	tctacagcggccgcgagctctctttgaaaagataatgtat

**R2_KpnI_gBlock**	TATAGAGCGGCCGCGGTACCAGACATAAAAAACAAAAAAAG
**R1_ADH1_gBlock**	ACTTACCGTGGGATTCGTAGGATCATTTATCTTTCACTGCG
**F2_ADH1_gBlock**	ctacgaatcccacggtaagtgttttagagctagaaatagcaagt
**F_ADH1(noxE)_repair**	GCACAATATTTCAAGCTATACCAAGCATAC
**R_ADH1(noxE)_repair**	AACCTAAGAGTCACTTTAAAATTTGTATACAC

Table S2. Synthesis of the guideRNA-expressing cassettes used in this study.

	Sequences
**gRNA_dPDC1**	TCTACAGCGGCCGCGAGCTCTCTTTGAAAAGATAATGTATGATTATGCTTTCACTCATATTTATACAGAAACTTGAT<br>GTTTTCTTTCGAGTATATACAAGGTGATTACATGTACGTTTGAAGTACAACTCTAGATTTTGTAGTGCCCTCTTGGG<br>CTAGCGGTAAAGGTGCGCATTTTTTCACACCCTACAATGTTCTGTTCAAAAGATTTTGGTCAAACGCTGTAGAAG<br>TGAAAGTTGGTGCGCATGTTTCGGCGTTCGAAACTTCTCCGCAGTGAAAGATAAATGATCTTGTTCCAGACACGA<br>CGTCAGTTTTAGAGCTAGAAATAGCAAGTTAAAATAAGGCTAGTCCGTTATCAACTTGAAAAAGTGGCACCGAGT<br>CGGTGGTGCTTTTTTTGTTTTTTATGTCTGGTACCGCGGCCGCTCTATA
**gRNA_dPDC5**	TCTACAGCGGCCGCGAGCTCTCTTTGAAAAGATAATGTATGATTATGCTTTCACTCATATTTATACAGAAACTTGAT<br>GTTTTCTTTCGAGTATATACAAGGTGATTACATGTACGTTTGAAGTACAACTCTAGATTTTGTAGTGCCCTCTTGGG<br>CTAGCGGTAAAGGTGCGCATTTTTTCACACCCTACAATGTTCTGTTCAAAAGATTTTGGTCAAACGCTGTAGAAG<br>TGAAAGTTGGTGCGCATGTTTCGGCGTTCGAAACTTCTCCGCAGTGAAAGATAAATGATCAACACCGTCTTCGGT

	TTGCCGTTTTAGAGCTAGAAATAGCAAGTTAAAATAAGGCTAGTCCGTTATCAACTTGAAAAAGTGGCACCGAGT<br>CGGTGGTGCTTTTTTTGTTTTTTATGTCTGGTACCGCGGCCGCTCTATA
**gRNA_dPDC6**	TCTACAGCGGCCGCGAGCTCTCTTTGAAAAGATAATGTATGATTATGCTTTCACTCATATTTATACAGAAACTTGAT<br>GTTTTCTTTCGAGTATATACAAGGTGATTACATGTACGTTTGAAGTACAACTCTAGATTTTGTAGTGCCCTCTTGGG<br>CTAGCGGTAAAGGTGCGCATTTTTTCACACCCTACAATGTTCTGTTCAAAAGATTTTGGTCAAACGCTGTAGAAG<br>TGAAAGTTGGTGCGCATGTTTCGGCGTTCGAAACTTCTCCGCAGTGAAAGATAAATGATCCGTGAAACAGGCCG<br>TTGAGTGTTTTAGAGCTAGAAATAGCAAGTTAAAATAAGGCTAGTCCGTTATCAACTTGAAAAAGTGGCACCGAG<br>TCGGTGGTGCTTTTTTTGTTTTTTATGTCTGGTACCGCGGCCGCTCTATA

Figure S1. Fermentation of the *PDC* disrupted strains.

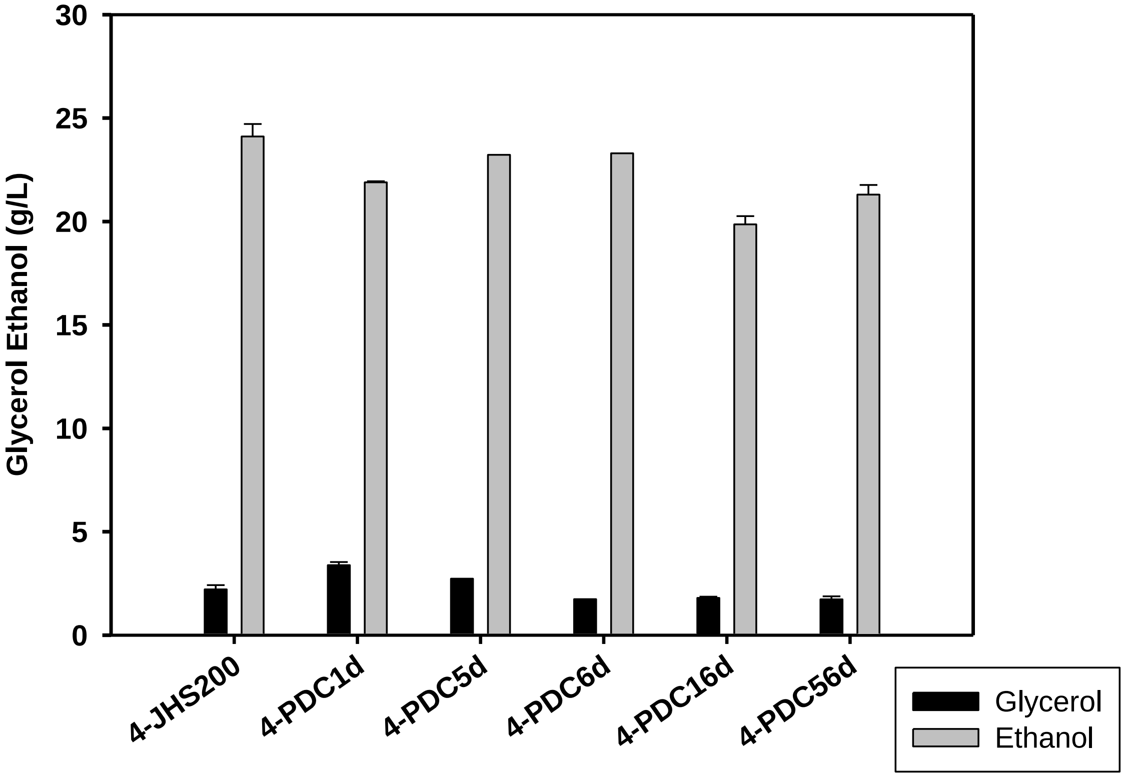

Figure S2. Fermentation profile of the YG01 strain.

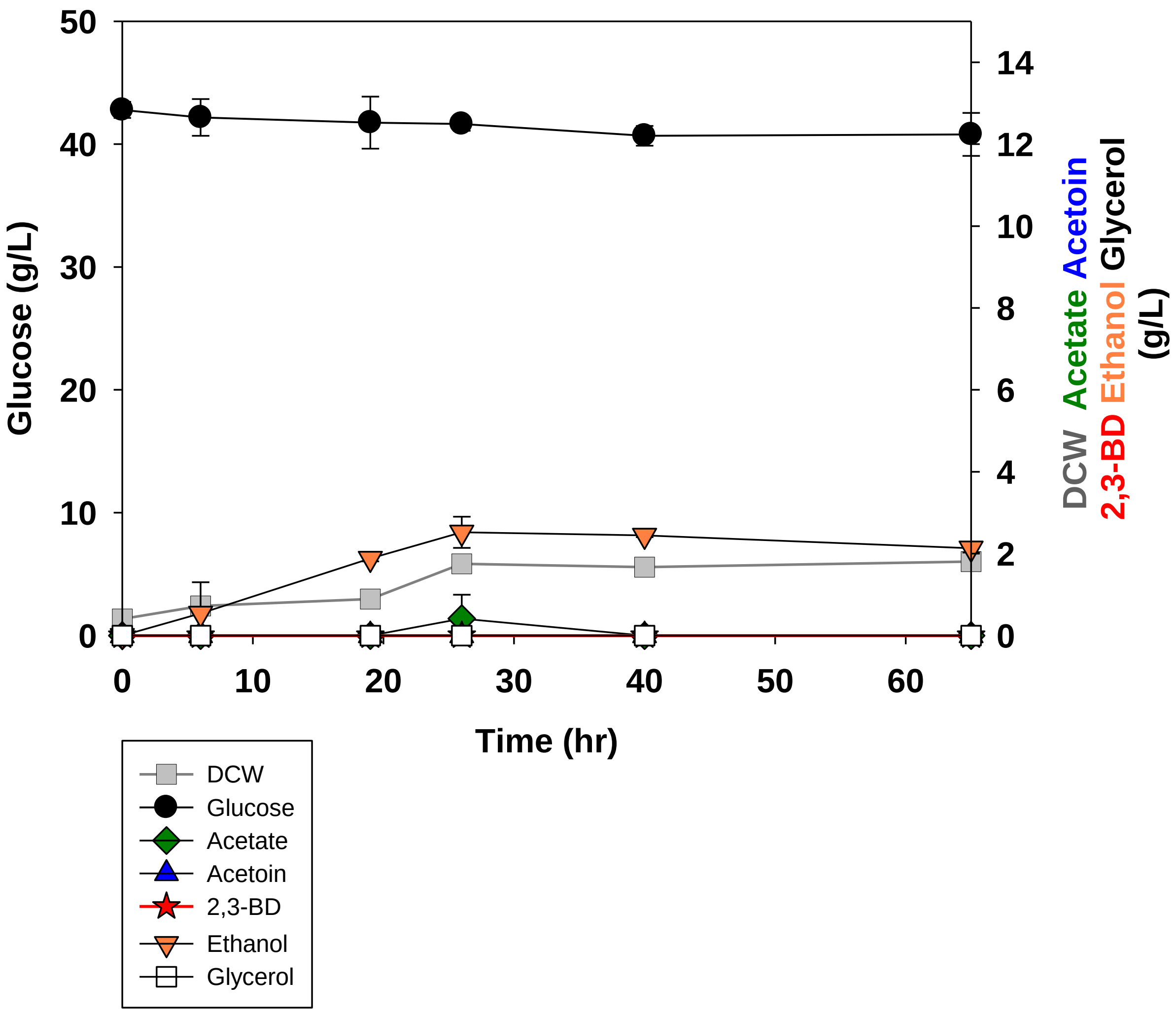

Figure S3. Fermentation profile of the YG02 strain.

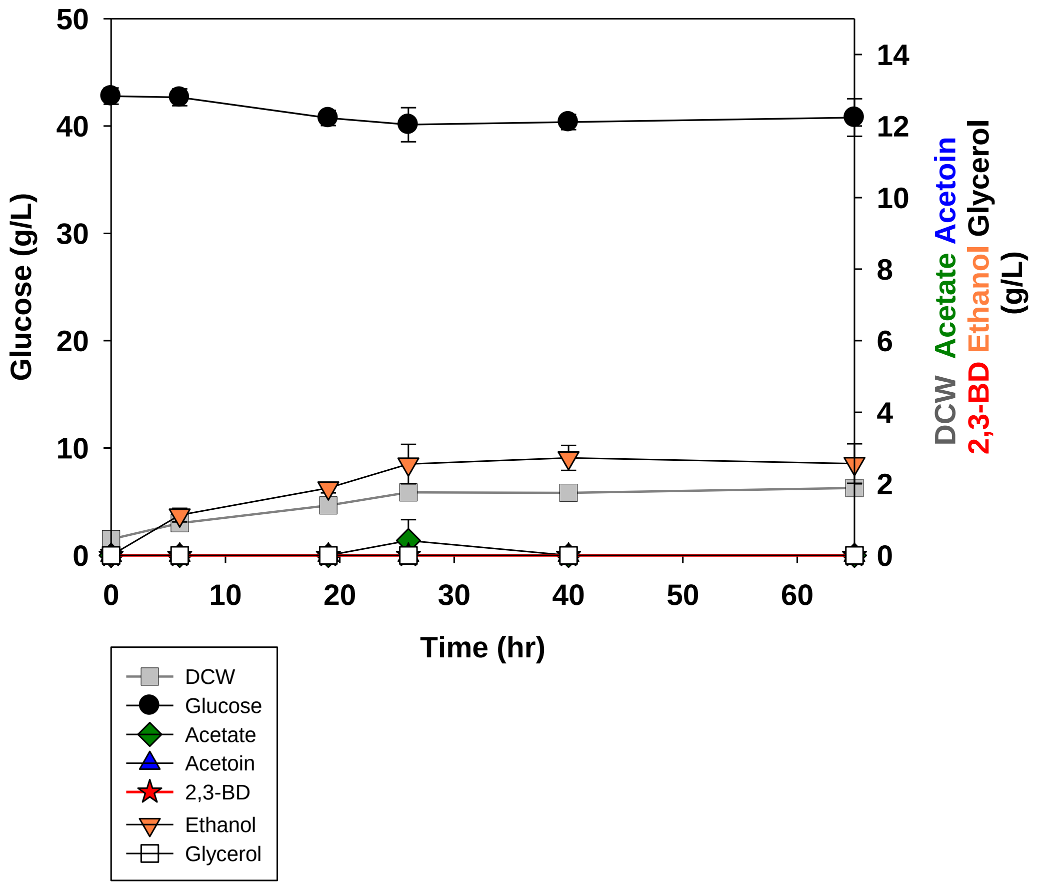

Figure S4. Confirmation of disrupting *PDC 1* and *6* in industrial polyploid *S. cerevisiae* after the several passage of cultivation.

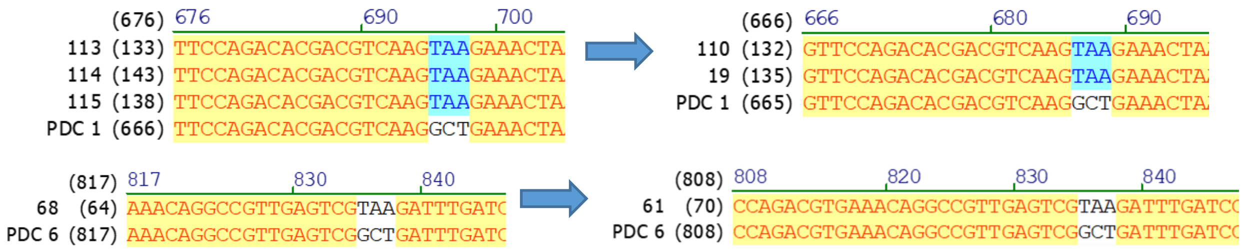

Supplement: Supplementary file 1 — Additional file 1. Additional tables and figures. [file 13068_2019_1545_MOESM1_ESM.pdf]
